# Identification of Intestinal NaCl Absorptive-Anion Secretory Cells: Potential Functional Significance

**DOI:** 10.3389/fphys.2022.892112

**Published:** 2022-07-19

**Authors:** Mark Donowitz, Rafiquel Sarker, Ruxian Lin, George McNamara, Chung Ming Tse, Varsha Singh

**Affiliations:** ^1^ Department of Medicine, The Johns Hopkins University School of Medicine, Baltimore, MD, United States; ^2^ Department of Physiology, The Johns Hopkins University School of Medicine, Baltimore, MD, United States

**Keywords:** NHE3, DRA, CFTR, Na absorption, anion secretion

## Abstract

Use of human enteroids studied in the undifferentiated and differentiated state that mimic the intestinal crypt and villus, respectively, has allowed studies of multiple enterocyte populations, including a large population of enterocytes that are transitioning from the crypt to the villus. This population expresses NHE3, DRA, and CFTR, representing a combination of Na absorptive and anion secretory functions. In this cell population, these three transporters physically interact, which affects their baseline and regulated activities. A study of this cell population and differentiated Caco-2 cells transduced with NHE3 and endogenously expressing DRA and CFTR has allowed an understanding of previous studies in which cAMP seemed to stimulate and inhibit DRA at the same time. Understanding the contributions of these cells to overall intestinal transport function as part of the fasting and post-prandial state and their contribution to the pathophysiology of diarrheal diseases and some conditions with constipation will allow new approaches to drug development.

## Introduction

### Intestinal Cell Populations Containing NHE3, DRA, and CFTR

The intestinal brush border Na^+^/H^+^ exchanger NHE3 (SLC9A3) accounts for the majority of intestinal Na^+^ absorption in the period between meals (Zachos et al., 2005). NHE3 is one of the most regulated transport proteins. It exchanges one Na^+^ for one H^+^ and is active under baseline or fasting conditions, while it is acutely inhibited in the early post-prandial state, presumably to contribute to the spreading of digestive enzymes over the intestinal surface, while later after eating it is acutely stimulated, presumably to prevent dehydration by reabsorbing luminal Na^+^/fluid (Zachos et al., 2005; Zachos et al., 2009a; Kiela and Ghishan, 2009; Ghishan and Kiela, 2012; Donowitz et al., 2013). Physiologically, it seems that its major function is to be inhibited as part of the digestive process. Multiple ligands both stimulate and inhibit NHE3, which include luminally released products of digestion, intestinal neurohumoral substances, systemically released hormones, and agents involved in pathologic intestinal changes that include bacteria, viruses, and parasites, among others. The acute regulation of NHE3 is primarily by changes in its trafficking between the brush border and endosomal system, with some of the effects involving phosphorylation and ubiquitination (Janecki et al., 1998; Kurashima et al., 1998; Collazo et al., 2000; Donowitz et al., 2005; Cha et al., 2006; Donowitz and Li, 2007; Donowitz et al., 2009; Hendus-Altenburger et al., 2014; Singh et al., 2014; Yang et al., 2014; Cha et al., 2017; Sarker et al., 2017; Pedersen and Counillon, 2019). This regulation involves multiprotein complexes that form on the NHE3 C-terminus, the composition of which changes as NHE3 traffics between the endosomal system and brush border and which is further altered as part of acute regulation of NHE3 (Yun et al., 1997; Weinman et al., 2000; Akhter et al., 2002; Cha et al., 2004; Li et al., 2004; Cha et al., 2006; Sarker et al., 2008; Zachos et al., 2008; Zachos et al., 2009b; Sarker et al., 2011; Zhu et al., 2011; Zizak et al., 2012; Zachos et al., 2013; Cha et al., 2014; He et al., 2015; He et al., 2016; Sarker et al., 2017). The components of these complexes have only been partially defined, and the association of NHE3 with other transport proteins as part of acute regulation has not been well-characterized. Predominantly, in the ileum and proximal colon, NHE3 is part of a transport process called electroneutral NaCl absorption in which NHE3 transport activity occurs in parallel with a brush border Cl^−^/HCO_3_^−^ exchanger of the SLC26A family, that is generally believed to be SLC26A3 (Downregulated in Adenoma, DRA) (Zachos et al., 2005; Zachos et al., 2009a; Kiela and Ghishan, 2009; Ghishan and Kiela, 2012; Donowitz et al., 2013).

The intestinal epithelial cells in which human NHE3 exists are generally believed to be the villus of the small intestine and surface cells of the proximal colon, while NHE3 is not thought to be present in the crypt population of both the small intestine and colon (In et al., 2016a). The mammalian intestine takes part in salt homeostasis by both actively absorbing Na^+^ and Cl^−^ and secreting Cl^−^ and HCO_3_^−^ (Barrett and Keely, 1999). The textbook models of these processes indicate that there are separate cells involved in absorption and secretion: the villus of the small intestine and surface cells of the colon carrying out Na^+^ absorption and the crypt cells of both the small intestine and colon carrying out active anion secretion, primarily contributed by the CFTR Cl^−^ channel. Since the crypt cells evolve into the villus cells over less than 4–5 days in humans, it has always been challenged about how these processes could be so distinct since so many different transport processes were involved in absorption and secretion. For this model to be correct, it was acknowledged that the transition required the absolute turning on and off expression of multiple transport proteins (Field, 2003).

These gaps in understanding were recently closed by the use of a) detailed IF examination of the human intestine with antibodies specific for NHE3, DRA, and CFTR and examining tissue arrays that allowed simultaneous evaluation of a large number of normal human intestinal tissue samples (Tuner, 2020) and b) using human enteroids in which crypt-like and villus-like populations could be created by altering the growth factors present (Sato et al., 2009; Sato et al., 2011; In et al., 2016b; Yu et al., 2017). These approaches revealed that throughout the small intestine and colon, there are populations of cells that are transitioning between the crypt and upper villus, with individual cells containing NHE3, DRA, and CFTR (Barrett, 2018; Yin et al., 2018; Tse et al., 2019). Using human enteroids, there is evidence that these cells are capable of carrying out both Na^+^ and Cl^−^ absorption along with electrogenic anion secretion. This cell population has been studied in human ileal and proximal colonic enteroids and in histologic sections of the human ileum and proximal colon. Moreover, it appears that NHE3, DRA, and CFTR physically and functionally interact in this cell population, which has implications for their function. Further understanding of the regulation of NaCl absorption and its component NHE3 and DRA and anion secretion by this cell population is needed to fully understand intestinal salt transport under physiologic baseline and post-prandial conditions and the changes that characterize diarrheal diseases. This new understanding has implications related to both normal physiologic regulation of intestinal salt transport and pathophysiologic transport that occurs in diarrheal diseases.

## Methods

### Cells and Antibodies

Cells used: Caco-2-BBe, Caco-2-BBe/HA_3_-NHE3, and Caco-2-BBe/DRA-KO cells were derived from cells provided by M. Mooseker and J. Turner (Sarker et al., 2017). Antibodies used: DRA antibody: mouse monoclonal antibody SLC26A3 (H8), Cat#: SC-376187, Santa Cruz Biotechnology. INC. NHE3 antibody: rabbit polyclonal antibody, Cat#: NBP1-82574, Novus Biologicals. CFTR antibody: mouse monoclonal antibody, Ab217, CF foundation. Flag antibody: mouse monoclonal antibody, Cat#: F3165, Sigma. GAPDH antibody: mouse monoclonal antibody, Cat#: G8795, Sigma. B-actin antibody: mouse monoclonal antibody, Cat#: A2228, Sigma. HA antibody: Clone 16B12 monoclonal antibody, Cat#: MMS-101P, Biolegend. Tenanapor was provided by Ardylex, Inc.

### Measurement of NHE3 Activity

NHE3 activity in Caco-2-BBe and Caco-2-BBe/DRA-KO cells expressing HA-NHE3 was determined fluorometrically using the intracellular pH-sensitive dye BCECF-AM, as described previously (Cha et al., 2017; Yin et al., 2018). Filter-grown cells were infected with Ad-HA-NHE3 on day 12 after reaching confluence, and 48 h later, the cells were serum-starved for at least 4 h before NHE3 activity was determined. HOE-694 (50 µM) was included in TMA and Na solutions to inhibit potential contributions of NHE1, NHE2, and NHE8 to NHE activity measured. The TMA solution contains 138 mM tetramethylammonium chloride (TMA-Cl), 5 mM KCl, 2 mM CaCl_2_, 1 mM MgSO_4_, 1 mM NaH_2_PO_4_, 25 mM glucose, 20 mM HEPES, and pH 7.4; whereas the Na solution contains 138 mM NaCl, 5 mM KCl, 2 mM CaCl_2_, 1 mM MgSO4, 1 mM NaH_2_PO4, 25 mM glucose, 20 mM HEPES, and pH 7.4. The cells were loaded for 20 min at 37°C with 10 µM BCECF-AM in 50 mM NH_4_Cl solution (98 mM NaCl, 5 mM KCl, 2 mM CaCl_2_, 1 mM MgSO4, 1 mM NaH_2_PO_4_, 25 mM glucose, 20 mM HEPES, and 50 mM NH_4_Cl, pH 7.4). The filters were then mounted in a cuvette, placed in a fluorometer (Horiba-Photon Technology, Lawrenceville, NJ), and perfused from both sides with TMA medium to rapidly remove NH_4_^+^ to acidify the intracellular space. After 2–3 min, the apical TMA medium was replaced with Na medium for Na-dependent pHi recovery. For each cell monolayer on filters, pHi was calibrated using K-clamp solutions set at pH 6.0, 6.6, and 7.3 with 10 μM nigericin. Data were analyzed using Origin 8.0 software (OriginLab, Northampton, MA, United States). Initial rates of Na-dependent intracellular alkalinization were calculated for a given pHi over the first 1 min of Na exposure and are expressed as ∆pH/∆t.

### Measurement of Cl^−^/HCO_3_^−^ Exchange Activity

Caco-2-BBe cells were seeded onto 12-well Transwell inserts for 12–14 days and were infected with adeno-HA-NHE3 or empty adenoviral vector (without NHE3). Forty-eight hours after viral infection, the cells were serum-starved for at least 4 h, and Cl^−^/HCO_3_^−^ exchange activity was measured fluorometrically using the pHi-sensitive dye BCECF-AM and a custom chamber allowing separate apical and basolateral superfusion, as previously described (Yin et al., 2018). The cells were incubated with 10 μmol/L BCECF-AM in Na solution (138 mmol/L NaCl, 5 mmol/L KCl, 2 mmol/L CaCl_2_, 1 mmol/L MgSO_4_, 1 mmol/L NaH_2_PO_4_, 10 mmol/L glucose, 20 mmol/L HEPES, and pH 7.4) for 20–30 min at 37°C and mounted in a fluorometer (Horiba-Photon Technology, Lawrenceville, NJ). The basolateral side of Caco-2-BBe cells was superfused continuously with Cl^−^ solution, whereas the apical surfaces of cells were superfused with Cl^−^ solution (110 mmol/L NaCl, 5 mmol/L KCl, 1 mmol/L CaCl_2_, 1 mmol/L MgSO_4_, 10 mmol/L glucose, 25 mmol/L NaHCO_3_, 1 mmol/L amiloride, 5 mmol/L HEPES, and 95% O_2_/5% CO_2_) or Cl^−^-free solution (110 mmol/L Na-gluconate, 5 mmol/L K-gluconate, 5 mmol/L Ca-gluconate, 1 mmol/L Mg-gluconate, 10 mmol/L glucose, 25 mmol/L NaHCO_3_, 1 mmol/L amiloride, 5 mmol/L HEPES, and 95% O_2_/5% CO_2_) under a flow rate of 1 ml/min. The transition between Cl^−^-solution and Cl^−^-free solution causes HCO_3_^−^ movement across the cell membrane performed by Cl^−^/HCO_3_^−^ exchanger(s), and the resulting change in pHi was recorded. Two cycles of removing/replenishing extracellular Cl^−^ were performed to determine the Cl^−^/HCO_3_^−^ exchange activity under basal conditions. At the end of each experiment, pHi was calibrated using K-clamp solutions with 10 μmol/L nigericin that were set at pH 6.8 and 7.8. The rate of initial alkalinization after the transition from Cl^−^ solution to Cl^−^-free solution was calculated as DRA activity using Origin 8.0 software (OriginLab, Northampton, MA, United States). Initial rates of Cl-removal–dependent intracellular alkalinization were calculated for a given pHi over the first 1 min of Cl -removal and are expressed as ∆pH/∆t.

### Immunoblotting

The cells were rinsed three times with phosphate-buffered saline and harvested in phosphate-buffered saline by scraping. Cell pellets were collected by centrifugation, solubilized in lysis buffer containing a protease inhibitor cocktail, and homogenized by sonication as described before (Yin et al., 2018). Protein concentration was measured using the bicinchoninic acid method. Proteins were incubated with sodium dodecyl sulfate buffer (5 mmol/L Tris-HCl, 1% sodium dodecyl sulfate, 10% glycerol, 1% 2-mercaptoethanol, pH 6.8) at 37°C for 10 min, separated by sodium dodecyl sulfate-polyacrylamide gel electrophoresis on a 10% acrylamide gel, and transferred onto a nitrocellulose membrane. The blot was blocked with 5% nonfat milk; probed with primary antibodies against DRA (mouse monoclonal, 1:500, sc-376187; Santa Cruz), CFTR [mouse monoclonal, 1:300, Cystic Fibrosis Foundation Therapeutics (Chapel Hill, NC)], NHE3 (rabbit polyclonal, NBP1, 1:100, Novus (Littleton, CO)), glyceraldehyde-3-phosphate dehydrogenase (mouse monoclonal, 1:5000, G8795; Sigma-Aldrich), β-actin (mouse monoclonal, 1:5000, A2228; Sigma-Aldrich) overnight at 4°C; and followed by secondary antibody against mouse IgG (1:10,000) for 1 h at room temperature. Protein bands were visualized and quantitated using an Odyssey system and Image Studio software (LI-COR Biosciences, Lincoln, NE).

### Immunofluorescence Microscopy

The cells were fixed in 4% paraformaldehyde for 40 min, incubated with 5% bovine serum albumin/0.1% saponin in phosphate-buffered saline for 1 h, and incubated with primary antibody against DRA (mouse monoclonal, 1:100, sc-376187; Santa Cruz, Dallas, TX, United States) or CFTR (rabbit polyclonal, 1:200, ab131553, Abcam) overnight at 4°C. The cells then were incubated with Hoechst 33342 and secondary antibody against mouse IgG (1:100) for 1 h at room temperature. Finally, the cells were mounted and studied using a Carl Zeiss LSM510/META confocal microscope (Thornwood, NY).

### Super Resolution Microscopy

The cells were fixed with 4% paraformaldehyde and blocked and incubated with primary FLAG-mab and NHE3-pab with 1% BSA + 0.1% saponin in PBS. Abberior STAR RED goat anti-mouse and Abberior STAR orange goat anti-rabbit secondary antibodies were used to label FLAG-DRA and NHE3, respectively. The cell membranes were cut out and placed with the apical side down in a glass-bottom dish with Abberior antifade mount solution.

Stimulated Emission Depletion (STED) super-resolution microscopy: STED was acquired with STEDycon scanhead (Abberior) on a Nikon 100x/1.40 NA objective lens on a Ti2 inverted microscope, whose specifications are as follows: pixel size 25 nm, with typical field of view 16 × 16 um and pixel dwell time 10 µsec, with 5-line accumulation (avalanche photodiode photon-counting detector), along with 775 nm depletion laser wavelength, 561 nm excitation for Abberior STAR Orange, 640 nm excitation for Abberior STAR Red, and time gate 1–7 ns (40 MHz laser pulses). Shown are single XY sections (600 µM thick) at the outermost apical membrane.

### Quantitative PCR

Total RNA was prepared from cultured cells using TRIzol (Invitrogen) according to the manufacturer’s protocol. One microgram of RNA was used for reverse transcription with the iScript cDNA synthesis kit (Bio-Rad). Real-time PCR was performed (iQ SYBR Green Supermix, Bio-Rad) on cDNA using primers used before (Yin et al., 2018). The relative fold changes in mRNA levels of NHE3, CFTR, and DRA between differentiated enteroids and undifferentiated enteroids (set as 1) were determined using the 2 ^−ΔΔCT^ method with human *18S* ribosomal RNA simultaneously studied and used as the internal control for normalization.

### Proximity Ligation Assay (PLA)

PLA was performed according to the manufacturer’s instructions (Olink). Fifteen days post confluence, Caco-2-BBE cells were treated with Fsk followed by fixing in 4% PFA for 1 h, followed by the blocking step as per the manufacturer’s instructions. The cells were incubated with mouse DRA and rabbit CFTR or rabbit NHE3 primary antibodies overnight at 4°C, followed by incubation with secondary antibodies conjugated with the PLA probe at 37°C for 1 h as recommended by the manufacturers. Then, ligation and amplification were performed (Duolink detection kit Orange, 555 nm). Finally, a mounting medium with 4,6-diamidino-2-phenylindole (DAPI) was used. The cells were scanned using an FV3000 confocal microscope. Four non-overlapping fields of view per well were identified, and photomicrographs were acquired under each experimental condition. The images were acquired using ×20 or ×40 oil immersion objective on an FV3000 confocal microscope (Olympus, Tokyo, Japan) with software (Olympus) and processed with NIH ImageJ. To discriminate PLA puncta from the background fluorescence, identical for all conditions, the manually selected threshold was applied to all images. The number of nuclei (DAPI+; ∼170 per field of view per condition) and total puncta (red spots) was counted using the Duolink1 Image Tool Software (Olink Bioscience) from each of the four fields of view and averaged for each experimental condition for statistical comparison, with a total of five biological replicates; a one-way ANOVA followed *by a priori* comparisons with Tukey’s test or Student’s t-test was conducted, as appropriate.

## Results

### NHE3, DRA, and CFTR are all Expressed in an Upper Crypt Enterocyte Population

**FIGURE 1:**
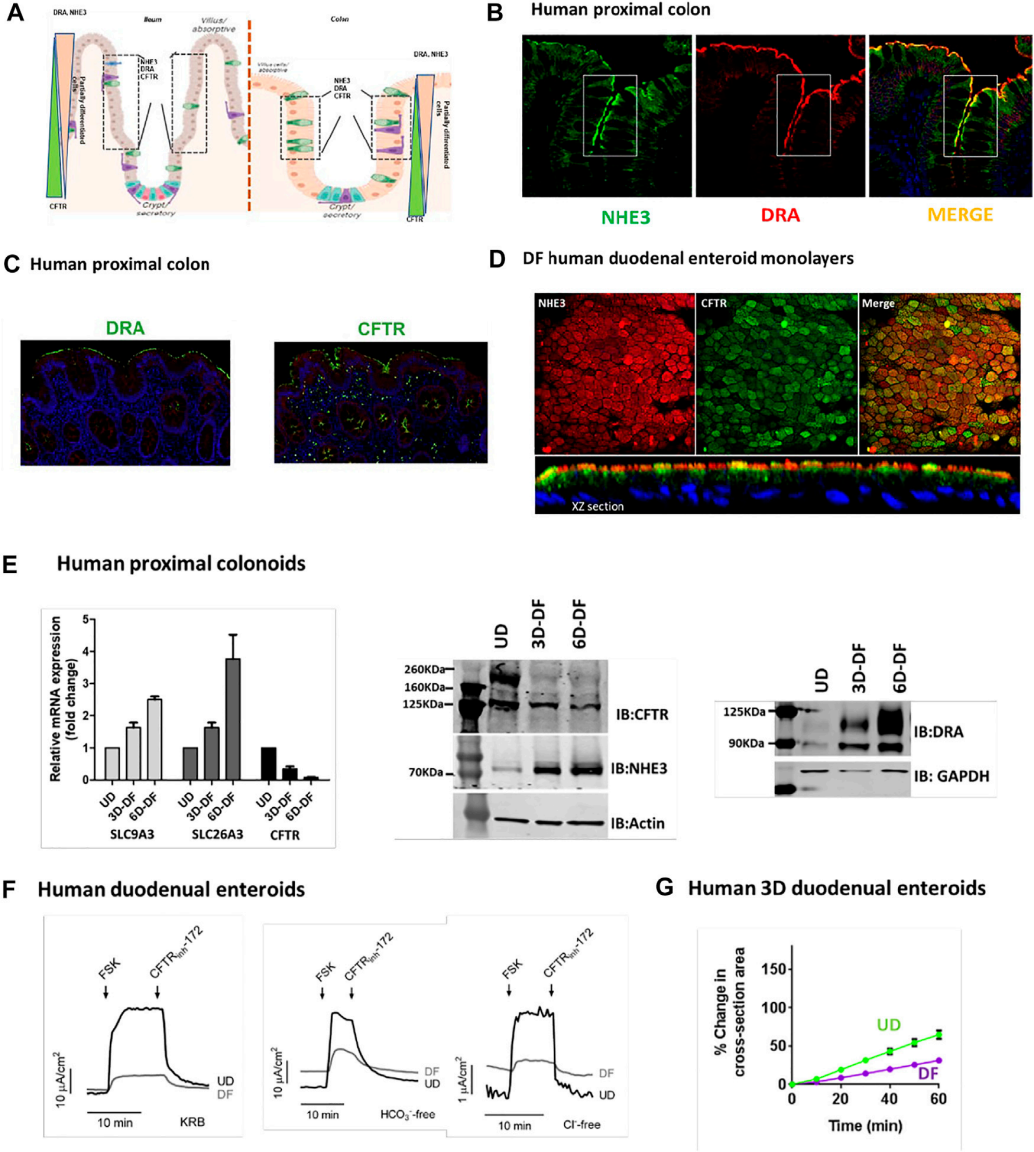
**(A)**. Cartoon showing localization of ileal and colonic enterocytes expressing NHE3, DRA, and CFTR in the same cell. The boxed area shows the cell population in which NHE3, DRA, and CFTR occur in the same cells. **(B)**. Immunofluorescence of the human proximal colon shows the overlap in the distribution of NHE3 and DRA [from Atlas of Intestinal Transport (Tuner, 2020)]. **(C)**. Representative immunofluorescence of the human proximal colon showing localization of DRA and CFTR in the same cell populations [from (Tse et al., 2019)]. **(D)**. Immunofluorescence of differentiated normal human duodenal enteroids (XY sections, above; XZ sections, below), demonstrating both NHE3 and CFTR in the same cells. NHE3 (red) is primarily in the BB, while CFTR is expressed in some cells in the BB and in some cells subapically. **(E).** Expression (mRNA, left; protein, middle and right) of NHE3, DRA, and CFTR in human proximal colonic enteroids comparing growth in an undifferentiated state (grown in Wnt3A, R-spondin, and noggin) and when differentiated by removal of Wnt3A and R-spondin for 3 days (“partially differentiated”) or 6 days (“differentiated”). **(F)**. CFTR-dependent active anion secretion is present in both undifferentiated and differentiated normal human duodenal enteroid monolayers. Short-circuit current of voltage-clamped duodenal enteroid monolayers is shown in response to forskolin (10 µM). Active anion secretion occurs in both the undifferentiated and differentiated enteroids (undifferentiated > differentiated), and the secretion occurs both in the absence of Cl^−^ or HCO_3_^−^ in the bathing solutions. All the demonstrated anion secretion is CFTR-dependent on being inhibited by CFTR_inhibitor_-172 [from (Yin et al., 2018), Figures 7A–C]. **(G)**. Forskolin-induced swelling assay in normal human 3D duodenal enteroids occurred in both differentiated and undifferentiated states (undifferentiated > differentiated) [from (Yin et al., 2018), Figure 5J].

The cell population expressing NHE3, DRA, and CFTR based on IF studies of tissue arrays from the Atlas of Intestinal Transport (Tuner, 2020) in human ileum includes the upper crypt and lower villus and in human proximal colon upper crypt and surface cells, although some differences in location of expression were present among individual normal subjects (Cartoon Figure 1A ileum and colon). As examples of co-localization in the same cell population, we performed additional studies: Figure 1B shows IF of NHE3 and DRA in the human proximal colon, and Figure 1C shows DRA and CFTR in the same cells, also in the human proximal colon. Figure 1D shows differentiated human duodenal enteroids expressing NHE3 and CFTR in the same cells. These cells were further characterized in small intestinal and colonic enteroids comparing undifferentiated enteroids grown in Wnt 3A, R-Spondin, and noggin with the same enteroids grown for 3 and 6 days in the absence of Wnt and R-Spondin. qRT-PCR for human proximal colonoids is shown with message and protein for NHE3, DRA, and CFTR, changing with the state of differentiation, but at 3 days of Wnt withdrawal-induced differentiation, NHE3, DRA, and CFTR message and protein are present, with the DRA protein very minimally present in UD and CFTR much reduced but present with 3 days of differentiation (Figure 1E).

Evidence for the function of NHE3 and DRA in differentiated small intestinal enteroids has been presented in multiple studies (In et al., 2016b; Yin et al., 2018; Tse et al., 2019). Functionally, the strongest evidence for the presence of CFTR in both undifferentiated (crypt-like) and differentiated (villus-like) small intestinal enteroids includes the demonstration that cAMP-stimulated anion secretion was present in both (Tse et al., 2019). This is shown in Figure 1F, both as forskolin-stimulated active anion secretion that was present both in the presence of both Cl^−^ and of HCO_3_^−^ and in the absence of each anion, studied separately, with the level of secretion paralleling the changes in CFTR expression. This is further demonstrated by forskolin-induced secretion, indicated by time-dependent swelling in 3D human duodenal enteroids, occurring in both differentiated and undifferentiated enteroids (Figure 1G).

**FIGURE 2:**
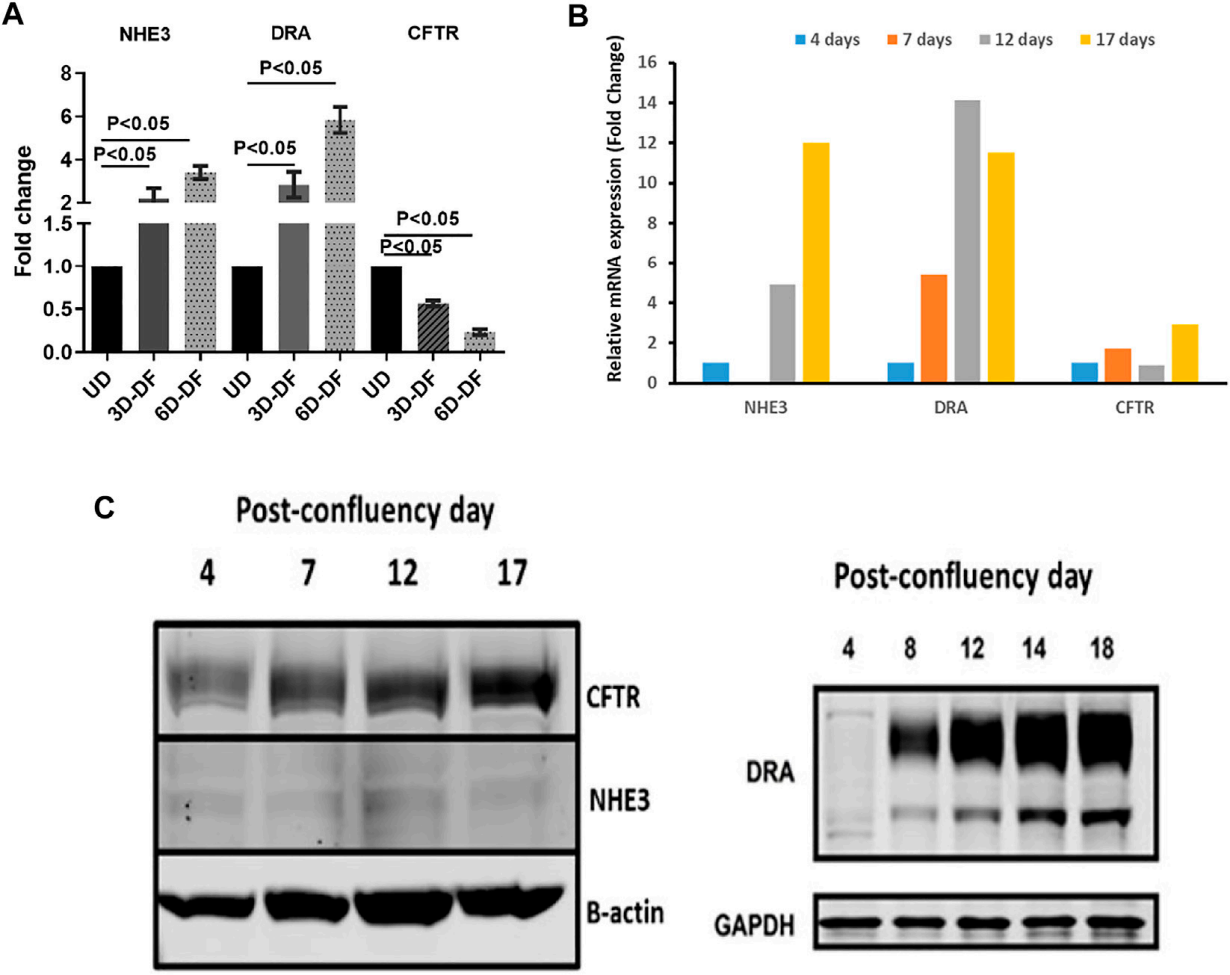
**(A)**. Differentiation-dependent protein expression of NHE3, DRA, and CFTR in human enteroids. *n* = 3 (paired t-tests). Similar time-dependent mRNA **(B)** and protein **(C)** expression of NHE3, DRA, and CFTR in Caco-2-BBe monolayers with days post confluency.

This cell population expressing NHE3, DRA, and CFTR can be modeled in both human enteroids and Caco-2-BBe cells based on days post confluency. Figure 2 shows that based on days of differentiation, the balance among NHE3, DRA, and CFTR changes, although all are expressed. While NHE3 was expressed, it was at a very low level even in differentiated Caco-2-BBe cells; and to study NHE3 function, stable transduction with lentiviral-NHE3 or transient transduction with adenoviral-NHE3 was used.

### Functional Effects of NHE3, DRA, and CFTR Being in the Same Cell

**FIGURE 3:**
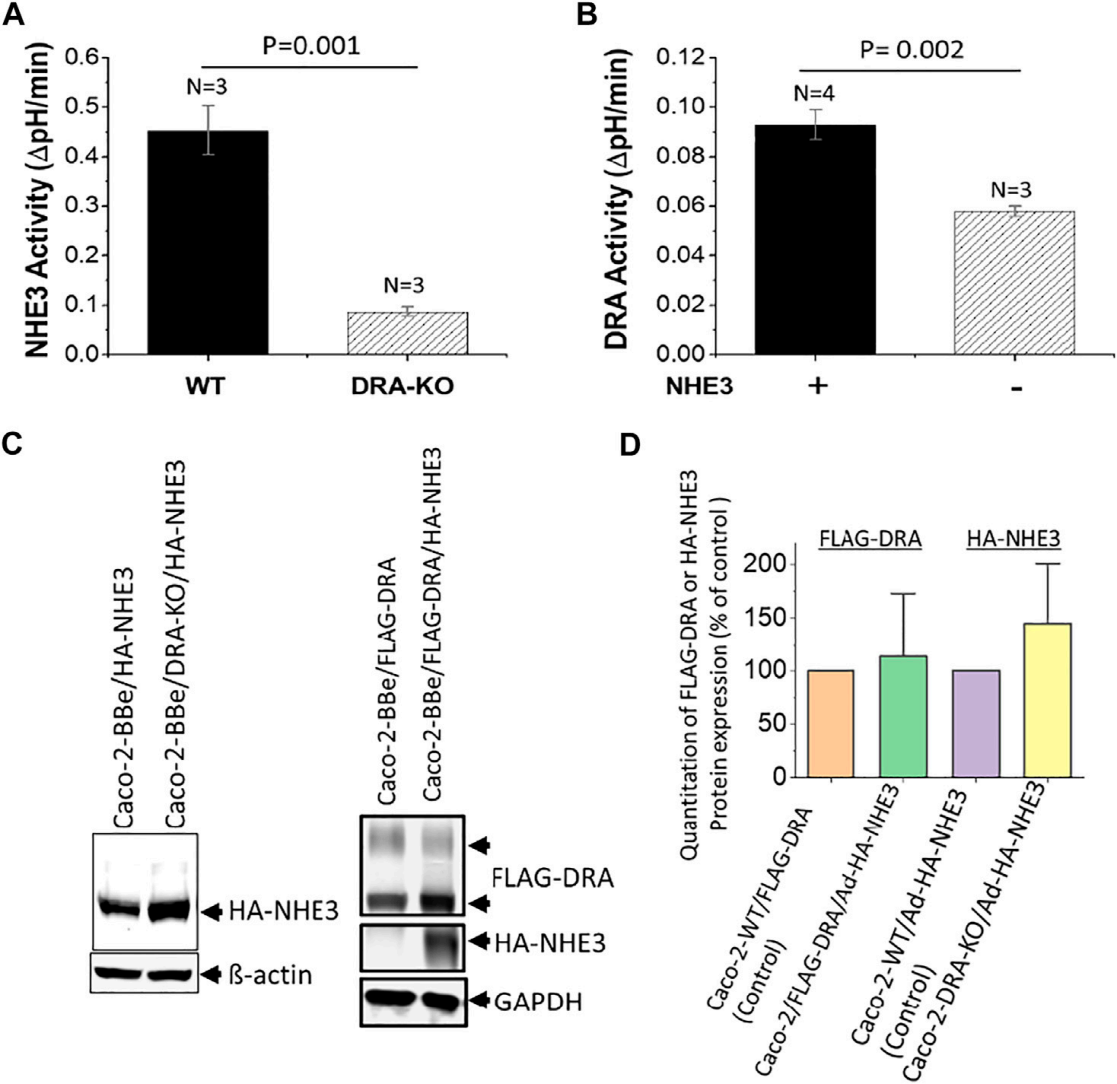
Reciprocal dependence of basal NHE3 and DRA activities in Caco-2-BBe cells. **(A)**. NHE3 activity is measured as a change in pH_i_ caused by apical Na^+^ addition to acutely NH_4_Cl^−^acidified cells expressing BCECF. Comparison is between cells in which DRA is KO by CRISPR/Cas9. n is the number of separate experiments. **(B)**. DRA activity is measured as apical Cl^−^ removal in presence of HCO_3_^−^-dependent alkalinization determined with BCECF. Comparison is between wild-type cells and those in which NHE3 is exogenously expressed by adenoviral transduction. n is the number of separate experiments. **(C)**. Western blots of NHE3 and DRA expression in the conditions shown in Figures 3A,B demonstrate that the cross-dependence of NHE3 and DRA was not dependent on their protein expression level. Single experiments are shown. **(D)**. Quantitation of Western blots from three experiments as in Figure 3C. The results are expressed as mean ± SEM. *n* = 3, and results are not significantly different than the individual controls.

There is evidence that NHE3, DRA, and CFTR interact in these cells to affect the regulation of each other. A complete evaluation of the co-dependence of these transporters has not been completed. Under baseline conditions, in Caco-2-BBe cells stably expressing NHE3, there is NHE3 activity measured as Na^+^-dependent, Tenapanor-sensitive alkalinization using the intracellular pH indicator BCECF. Similarly, using BCECF to measure intracellular pH, DRA is defined by alkalinization induced by Cl^−^ removal from the apical solution that contains HCO_3_^−^ and is inhibited by the DRA inhibitor, DRA_inhibitor_-250 (Tse et al., 2019). NHE3 and DRA activities, in part, depend on the presence of each other. Baseline NHE3 activity was significantly reduced in Caco-2-BBe cells in which DRA was KO using CRISPR/Cas9 (Figure 3A). Similarly, DRA activity was dependent on whether NHE3 was expressed in Caco-2-BBe cells (Figure 3B). This cross-dependence, however, was not related to the level of expression; that is, NHE3 protein expression was similar in the presence and absence of DRA, and DRA protein expression was similar in the presence and absence of NHE3 (Figure 3C). This indicates that this is a functional co-dependence. In addition, cAMP inhibits NHE3 and stimulates DRA and CFTR activities (Tse et al., 2019), although this has not been uniformly observed.

Importantly, Ko and Muallem previously showed that CFTR and DRA stimulated the transport of each other after cAMP, effects that required their physical interactions that involved the DRA STAS domain and the phosphorylated R domain of CFTR (Choi et al., 2001; Park et al., 2002; Ko et al., 2004; Shcheynikov et al., 2006a; Shcheynikov et al., 2006b). This dependence occurred only with a low concentration of the transporters, although the explanation was not provided experimentally. It was, however, suggested that binding of both proteins *via* their C-terminal type I PDZ domain recognition sequences made it likely that interactions with members of the NHERF family of scaffold proteins were involved.

**FIGURE 4:**
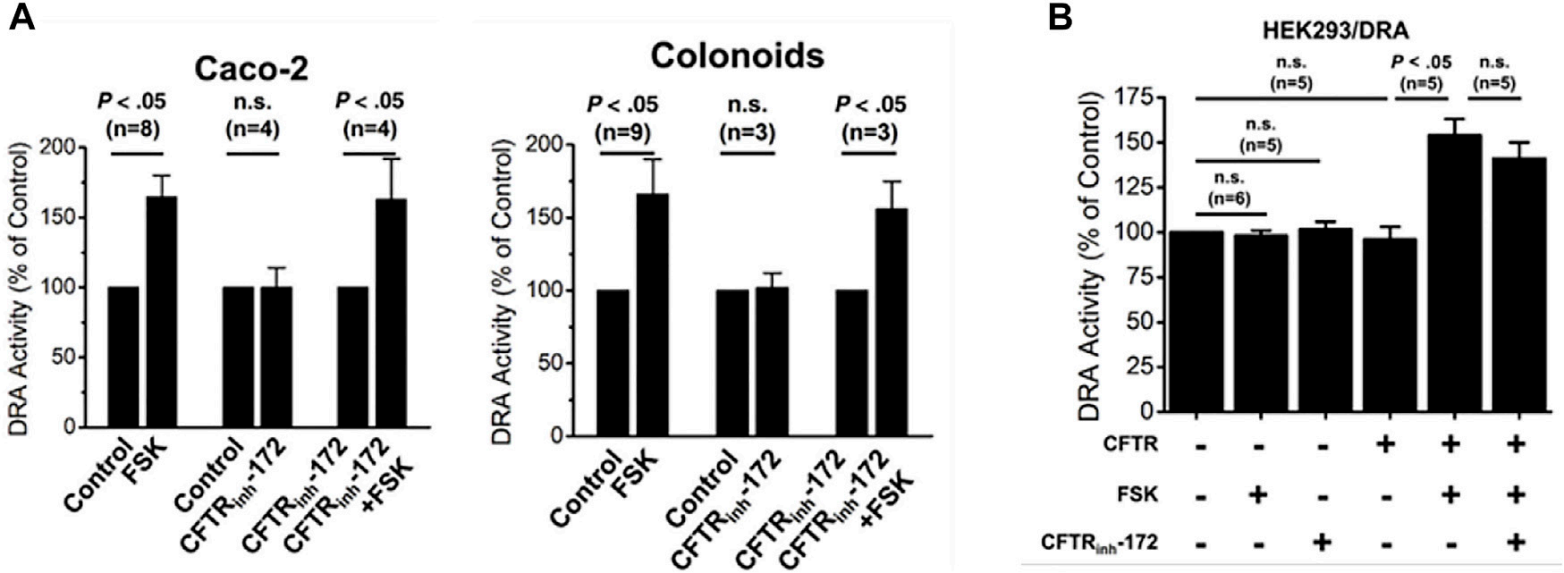
CFTR presence but not activity is necessary for cAMP stimulation of DRA in Caco-2-BBe cells, human colonoids, and HEK cells. **(A)**. In both Caco-2-BBe cells and human proximal colonoid monolayers, both cells express CFTR and forskolin-stimulated DRA activity which was not inhibited by the CFTR_inhibitor_-172 (from (Tse et al., 2019), Figs 8A and D, respectively). **(B)**. In HEK293 cells expressing DRA, forskolin (10 µM) stimulates DRA activity, but this only occurred when CFTR was expressed. This CFTR-dependent stimulation of DRA by cAMP was not altered by the CFTR_inhibitor_-172 (from (Tse et al., 2019), Figure 7A).

In differentiated human colonoid monolayers and Caco-2-BBe cells, cAMP stimulation of DRA activity was not altered in cells in which CFTR was inhibited by CFTR_inhibitor_-172 (Tse et al., 2019) (Figure 4A). While the effect of KO CFTR was not determined in these epithelial cells, in HEK293 cells, the presence of CFTR was necessary for cAMP stimulation of DRA (Tse et al., 2019) (Figure 4B). Together these results suggest that it is the presence of CFTR and not its activity that is involved in inducing cAMP stimulation of DRA.

### Mechanisms of NHE3, DRA, and CFTR Regulation in the Same Cells

**FIGURE 5:**
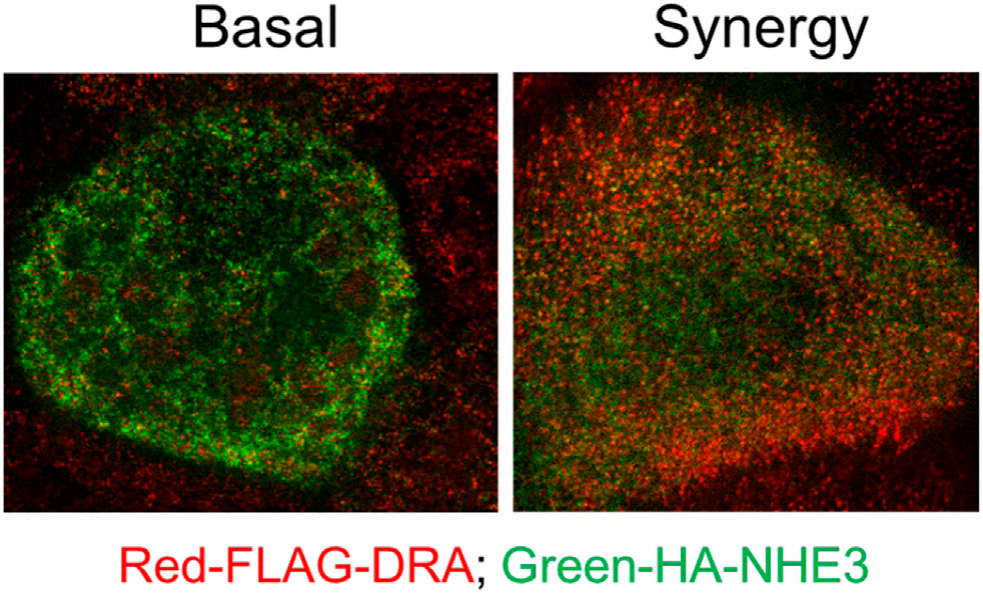
Super-resolution (STED) microscopy comparing relative BB localization of NHE3 and DRA under baseline conditions and after exposure to low concentrations of forskolin plus ATP which inhibits NHE3 and stimulates DRA activity. Immunostaining: Caco-2/FLAG-DRA stable cells grown in the 12-mm Transwell inserts were infected with adeno-HA-NHE3 and studied 48 h later. The cells were serum-starved for ∼4 h and treated on basolateral and apical surfaces with 1 µM FSK for 7 min and then with 0.25 µM ATP apically for 3 min. See methods for fixation, processing, and imaging details. This study shows there is more NHE3 than DRA on the outer BB under baseline conditions, but the opposite scenario is observed after exposure to forskolin plus ATP.

NHE3 is acutely regulated largely by changing rates of exocytosis/endocytosis. With cAMP, NHE3 inhibition is associated with less BB NHE3, which is seen in the accompanying super resolution figure in Caco-2-BBe cells expressing both NHE3 and DRA (Figure 5). DRA stimulation by cAMP involves both trafficking and functional activation. We reported an increase in surface DRA after cAMP indicated by cell surface biotinylation (Tse et al., 2019); however, the extent of stimulation exceeded the increase in surface expression (Tse et al., 2019). We conclude that cAMP stimulation of DRA involves not only trafficking but also direct activation without changing the amount of DRA on the BB.

**FIGURE 6:**
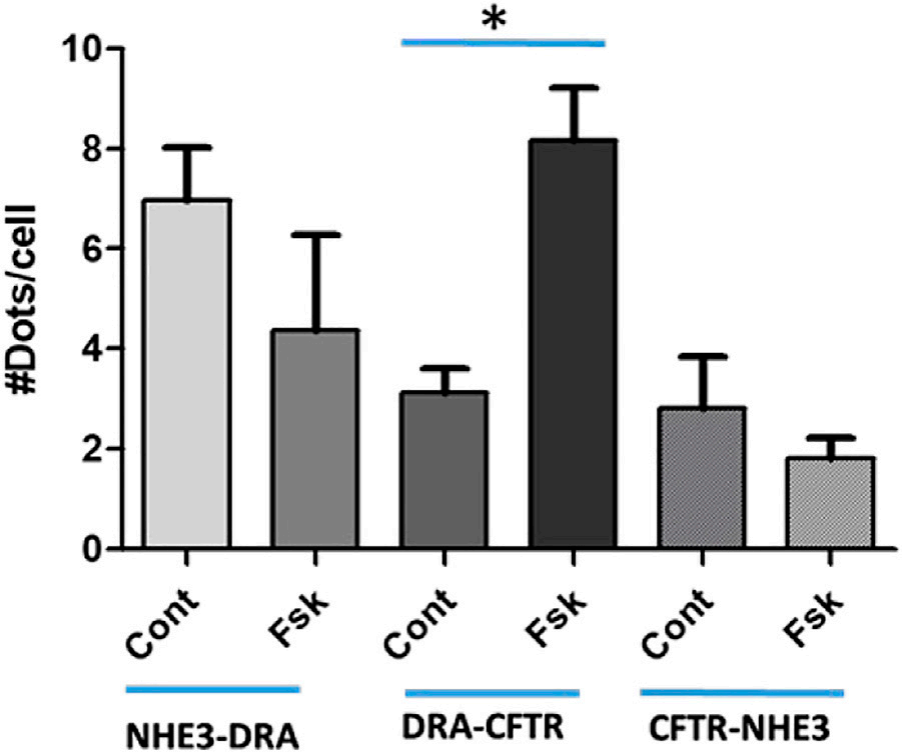
Proximity Ligation Assay (PLA) in Caco-2BBe cells shows direct binding under baseline conditions of NHE3-DRA, DRA-CFTR, and CFTR-NHE3. This binding was dynamic. Ten minutes after forskolin (10 µM) exposure, DRA-CFTR physical association was increased, while the physical association of NHE3-DRA and CFTR-NHE3 decreased. The results are expressed as means ± SEM of five experiments. *, *p* < 0.05.

Further mechanistic studies examined the physical association of NHE3, DRA, and CFTR after acute changes in transport with cAMP. Studies with proximity ligations assays (PLAs) (Figure 6) in polarized Caco-2-BBe cells showed that under basal conditions, there was a direct physical association of NHE3 with DRA, NHE3 with CFTR, and DRA with CFTR. Twenty minutes after forskolin exposure, the physical association between NHE3 and DRA and NHE3 and CFTR decreased while that between DRA and CFTR increased. The magnitude of the changes was largest for DRA and CFTR.

## Discussion

**FIGURE 7:**
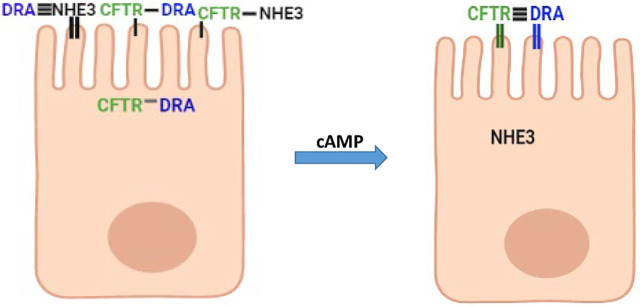
Suggested model in partially differentiated enterocytes of interactions of NHE3, DRA, and CFTR under baseline conditions and after cAMP. Horizontal lines connecting NHE3, DRA, and CFTR above microvilli indicate the extent of physical interaction (the more the horizontal lines, the greater the physical interaction). Vertical lines in microvilli associated with each protein indicate the percent of the total protein in the apical membrane (thick line high percentage).

The experimentally demonstrated presence of intestinal epithelial cells that represent a transitioning state from undifferentiated to fully differentiated state settles a long-standing debate concerning the relationship between intestinal cells taking part in Na^+^ absorption and anion secretion (Figure 1A). This sets the stage for studies to characterize by mRNA, proteomics, and functional methods the distribution in this cell population of a large number of transporter proteins present in the small intestine and colon that includes the basolateral and the apical plasma membrane and to define the functional role of this transitioning but large cell population. More specifically, the presence of NHE3, DRA, and CFTR in the same cells in a population of intestinal enterocytes explains, what appeared to be contradictory data, that there was less neutral NaCl absorption and increased Cl^−^ and HCO_3_^−^ secretion induced by secondary messengers including cAMP (Choi et al., 2001; Park et al., 2002; Ko et al., 2004; Shcheynikov et al., 2006a; Shcheynikov et al., 2006b; Musch et al., 2009). DRA associates more with NHE3 under baseline conditions and more with CFTR when cAMP is increased (Figure 7). In spite of the ability to perform unidirectional (Field, 2003) Cl^−^fluxes and intracellular Cl^−^ estimates, the balance between the contribution of DRA to apical Cl^−^ uptake as part of neutral NaCl absorption and to reuptake of the Cl^−^ secreted through CFTR and the stimulatory contribution to CFTR activity means that additional measurements are needed to allow interpretation and statement regarding which transport processes are quantitatively contributing to these Cl^−^ transport parameters.

The significance of this NaCl absorptive-anion secretory population of cells has only been partially characterized. Regulation by other secondary messengers that regulate NHE3, DRA, and CFTR and the contribution of these cells to intestinal diseases that involve transport of electrolytes, including diarrheal diseases and some diseases with constipation, including those with histologic damage to populations of enterocytes, are not yet evaluated. It is of interest that in some disorders, this population may be the major one affected with potential for changes in both magnitudes of NaCl absorption and anion secretion. Further studies are needed that deal with how to make use of the understanding of NHE3, DRA, and CFTR regulation in this cell population to deal with diarrheal diseases and constipation.
